# Graphene Oxide-Silver Nanoparticles in Molecularly-Imprinted Hybrid Films Enabling SERS Selective Sensing

**DOI:** 10.3390/ma11091674

**Published:** 2018-09-10

**Authors:** Yu Jiang, Davide Carboni, Luca Malfatti, Plinio Innocenzi

**Affiliations:** Laboratorio di Scienza dei Materiali e Nanotecnologie (LMNT), Dipartimento di Chimica e Farmacia, Università di Sassari, CR-INSTM, Via Vienna 2, 07041 Sassari, Italy; jyu@uniss.it (Y.J.); dcarboni@uniss.it (D.C.)

**Keywords:** molecular imprinting, graphene-mediated Surface-Enhanced Raman Scattering (G-SERS), organic-inorganic hybrid materials, Raman sensors

## Abstract

A highly sensitive and selective Raman sensor has been developed by combining molecularly imprinted cavities, silver nanoparticles, and graphene oxide into a hybrid organic-inorganic film. The molecular imprinted nanocomposite material is an advanced platform that exhibits Graphene-mediated Surface-Enhanced Raman Scattering. The sensing layers have been prepared via sol-gel process and imprinted with rhodamine 6G to obtain selective dye recognition. Graphene oxide sheets decorated with silver nanoparticles have been incorporated into the matrix to enhance the Raman scattering signal. The template molecule can be easily removed from the films by ultrasonication in ethanol. A 712-fold Raman enhancement has been observed, which corresponds to a 2.15 × 10^13^ count·μmol^−1^ signal enhancement per molecular cavity. Besides Raman enhancement, the sensing platform has shown an excellent selectivity toward the test molecule with respect to similar dyes. In addition, the material can be reused at least 10 times without any loss of performance.

## 1. Introduction

Metal nanoparticles with plasmonic properties, such as Ag, Au, and Cu, have been widely used as support for enhancing the Raman signal [1,2,3]. The enhancement factor (EF) of Surface Enhanced Raman Scattering (SERS)-active systems can be as high as 10^10^ due to the sum of the chemical and electromagnetic interactions between the analyte and the metal surface [4,5]. An enhanced Raman scattering has also been well documented for graphene [6,7]. In particular, the so-called Graphene-mediated-Enhanced Raman Scattering (GERS) has opened new perspectives for molecular detection based on Raman spectroscopy [8,9,10]. Graphene is much cheaper than the noble metal particles, it has good adsorption ability, and can quench the molecular fluorescence, which is typically detrimental for Raman characterization [11,12]. The EF in GERS-active platforms, however, is generally not higher than a factor of 100 because GERS is mainly due to a chemical mechanism. On the other hand, the high sensitivity of SERS systems is limited by the cost and low durability. Some nanoparticles, such as Ag, tend to be easily oxidized because of the local heating during SERS. In spite of the lower enhancement factor, GERS systems offer a better stability and absorption capability.

Several strategies have been explored to get more performing Raman sensors exploiting the potential of advanced SERS and GERS systems. For example, SERS and GERS composite platforms with porous structures have been developed. Our research group has prepared silica mesoporous films containing exfoliated graphene [13] or silver nanoparticles [14] as sensing platforms to achieve a selective detection of Rhodamine 6G (Rh6G), a molecule which can be considered as a benchmark for testing the properties of different SERS-active nanostructures. Moreover, some SERS composite platforms obtained by combining metal nanoparticles and maghemite particles (Fe_3_O_4_) have shown to improve the adsorption ability and to allow a rapid detection of Rh6G with a high reproducibility [15,16,17]. Additionally, a so-called Graphene-Surface Enhanced Raman Scattering (G-SERS) material has also been developed to increase the low sensitivity of GERS and the poor stability and adsorption ability of metal particles [18,19]. Generally, graphene oxide (GO) has been chosen to prepare the GO-Ag nanocomposite because the phenolic, carboxyl, and epoxide groups on the planes and edges of the GO surface give to GO itself good loading capabilities [20,21]. Different factors, however, affect the enhancement, such as the size, shape, and density of the Ag or Au nanoparticles attached to the GO surface [22,23,24,25]. Silica and titania mesoporous films containing exfoliate graphene and gold nanoparticles have been recently fabricated by our group and used as a 3D Raman sensing active platform to improve the detection sensitivity of test molecules [13]. All these G-SERS-active sensing materials have shown a better stability and sensitivity than GERS or SERS-active systems alone due to the combination of the electromagnetic mechanism from metal particles and chemical mechanism between the GO surface and the analyte. A wider application of these G-SERS sensing platforms is, however, limited by the poor selectivity.

Molecular imprinting works on the principle of “lock-and-key”, which can be explained as a way of making artificial “pore locks” for “molecular keys” [26,27]. This provides excellent molecular selectivity. The imprinted cavities inside the matrix can be built up by using a template molecule. After the template removal, the cavities in the matrix are used to recognize a specific molecule, which is a structural analogue of the molecular template. In general, molecularly imprinted materials cannot significantly enhance the Raman signal [28], and some other Raman active materials must be introduced, such as Ag, Au, or graphene derivatives. Chang et al. have designed a molecularly imprinted SERS-active platform by imprinting 4-mercaptobenzoic acid (4-MTA) onto the surface of Ag nanoparticles for selective detection of 4-MTA [29]. In our previous work, we have developed molecularly imprinted GERS-active platforms embedding exfoliated graphene. These sensing platforms have proved to have excellent selectivity and reproducibility [30,31,32]. A sol-gel silica matrix has been used for molecular imprinting instead of the more common organic polymers. The silica matrix, in fact, has a higher thermal stability, making the shape and size of the imprinted sites more stable, and avoiding damage under laser illumination during the Raman measurements. Although we have proved that the sensing platform containing exfoliated graphene and molecularly imprinted cavities can reach a five-fold enhancement of the Rh6G Raman signal with respect to a bare silica hybrid film, this enhancement is too low for sensing applications and needs to be improved before envisaging a practical application.

To increase the sensing performances, we have developed a nanocomposite hybrid material capable of merging molecular recognition properties and graphene-mediated surface enhanced Raman scattering. Specifically, molecularly imprinted silica hybrid films embedding GO-Ag nanocomposites have been designed as Raman-based sensing platforms (MI-G-SERS) for the detection of Rh6G.

## 2. Materials and Methods

### 2.1. Chemicals and Materials

Graphite (Sigma-Aldrich, Milan, Italy), cetyltrimethylammonium bromide (CTAB) (Sigma-Aldrich, 99%, Milano, Italy), ethanol (EtOH) (Fluka, >99.8%, Bucharest, Romania), silver nitrate (Sigma-Aldrich, 98% purity, Milan, Italia), hydrochloric acid (Sigma-Aldrich, 37% wt/wt, Milan, Italy), water (milli-Q, Milan, Italy), sodium citrate tribasic dehydrate (Sigma-Aldrich, 99% puritysodium borohydride ABCR, 98% purity, Karlsruhe, Germany), potassium manganate (VII) (Sigma-Aldrich, Milano, Italy), sodium hydroxide (Carlo Erba, Milan, Italy, 35% *w*/*v* in water), sulfuric acid (Fluka, 95–97% purity, Milano, Italy), orthophosphoric acid (Fluka, 85% purity, Bucharest, Romania), hydrogen peroxide (Alfa Aesar, 27%, *w*/*w*, Karlsruhe, Germany), rhodamine 6G chloride (Rh 6G) (Aldrich, 98% purity Milan, Italy), tetraethylorthosilicate (TEOS) (Aldrich, 99%, Milan, Italy), phenyltriethoxysilane (PhTES) (Aldrich, 98%, Milan, Italy), bis(triethoxysilyl)octane (B-TES-8) (Aldrich, 97%, Milan, Italy), methylene blue (MB) (Sigma, 98%, Milan, Italy), rhodamine B (Rh B) (Sigma, 97%, Milan, Italy), crystal violet (CV) (Aldrich, 98%, Milan, Italy). p-type boron doped, (100) cut, 381 mm-thick silicon wafers (Si-Mat, Kaufering, Germany, 1.5 × 1.5 cm^2^) were employed as substrates. Before use, the silicon wafers were washed with water, acetone, and ethanol, and then dried with compressed air. All the chemicals were used as received without further purification.

### 2.2. Preparation of Graphene oxide (GO) and Graphene Oxide-Silver Nanocomposite (GO-AgNPs)

Graphene oxide (GO) was prepared through a modified Hummers method [31]. The details of the preparation process are available in Appendix A. A final GO aqueous dispersion, with a concentration of 0.05 mg· mL^−1^ was obtained by sonication for 2 h. The GO-Ag nanocomposite was prepared by reduction of AgNO_3_ in the presence of GO. Briefly, 2 mL of AgNO_3_ solution (0.1 M) was added to 10 mL of GO aqueous dispersion and stirred for 30 min at room temperature. After that, 4 mL of sodium citrate tribasic dihydrate solution (0.1 M), used as a stabilizing agent, was added to the mixture. After stirring for 5 min at 60° C, 100 µL of a NaBH_4_ solution (0.1 M), used as a reducing agent, was added drop-wise. Afterwards, the pH was adjusted to 10 by a NaOH aqueous solution (0.2 M) and the mixture was then left reacting for 2 h at 60 °C. The resulting GO-AgNPs was centrifuged (10,000 rpm for 300 s) and washed by water for 6 times before being re-dispersed in ethanol.

### 2.3. Preparation of Molecular Imprinted (MIF) and not Imprinted (NIF) Films

8 mL of EtOH, 1.19 mL of Ph-TES, 1 mL of TEOS, 2.13 mL of B-TES-8, 0.30 mL of water, and 0.05 mL of hydrochloric acid (1 M) were mixed in a glass vial with the molar ratios, Ph-TES:TEOS:B-TES-8:EtOH:H_2_O:HCl = 1:1:1:30:6.4:0.025. After stirring for 5 min, 500 µL of CTAB ethanol solution (molar ratios TEOS:CTAB = 1:0.012; SiO_2_:CTAB = 1:0.004) were added to the solution and the resulting mixture was left to react under stirring for 2 h at 25 °C in a closed vial. 250 µL of GO-Ag ethanol dispersion were then added to 5 mL of the hybrid sol. After stirring for 30 min, the hybrid sol was divided into 2 parts; one half was mixed with 400 µL of Rh6G solution (0.1 M) and stirred for 2 h before depositing the molecularly imprinted composite films (MIF@GO-AgNPs) by spin-coating with a spinning rate of 1000 rpm for 40 s followed by 20 s at 500 rpm. 400 µL of ethanol were then added into the other half of the solution to reach the same overall volume as for the MIF@GO-AgNPs. The resulting solution was used to deposit the corresponding not imprinted film (NIF@GO-AgNPs) by following the same coating process as for MIF. A not imprinted hybrid film (NIF-H), to be used as a reference, was also prepared without adding Rh6G and the GO-Ag nanocomposite dispersion. Another reference sample was prepared by depositing a molecularly imprinted film without adding the GO-Ag nanocomposite dispersion (MIF-H). All of the films were kept for 20 h at 60 °C in an oven and then treated at 150 °C for 1 h.

### 2.4. Removal of the Molecular Template Rh6G

The molecularly imprinted films were washed with 3 mL of ethanol and ultrasonicated for 30 min (99% power, 400 VA, CP102, CEIA international, Paris, France) to remove the molecular template. The ethanol washing solutions were monitored by UV-Vis spectroscopy (Nicolet Evolution 300, Thermo Fisher, Waltham, MA, USA) after each step to evaluate the removal process. After 3 cycles of washing, the Rh6G was completely removed from the films and the overall concentration of Rh6G removed from each film was determined as the sum of the Rh6G removed during the 3 washing steps (Rh6G into 9 mL). The amount of Rh6G removed from the film was estimated by using a cross-dilution calibration curve that allowed an extinction coefficient for Rh6G at 517 nm (ε_517_ = 11890 L·mol^−1^·cm^−1^) to be obtained. The not-imprinted films were also washed by following the same procedure to make sure that all the samples underwent exactly the same treatment.

### 2.5. Characterization

UV-Vis spectroscopy. Optical absorption of samples was measured by a Nicolet Evolution 300 UV-Vis spectrophotometer (Thermo Fisher, Waltham, MA, USA) in the range of 200–800 nm with a bandwidth of 1.5 nm. Ethanol was used to correct the background absorption.

### 2.6. Fourier-Transform Infrared (FTIR) Spectroscopy

FTIR analysis was performed using infrared Vertex 70v interferometer (Bruker, Milan, Italy). The spectra were recorded in transmission mode between 4000 and 400 cm^−1^ by averaging 256 scans with 4 cm^−1^ of resolution. A silicon wafer was used as the background and the baseline was fitted by a concave rubber band correction with OPUS 7.0 software.

### 2.7. Transmission Electron Microscopy (TEM)

The TEM images were obtained using a TECNAI 200 microscope (FEI, Eindhoven, The Netherlands) working with a field emission electron gun operating at 200 kV. The samples were prepared by dispersing fragments of the films (obtained by stretching) onto a carbon-coated copper grid. GO-Ag nanocomposite fragments were dispersed in ethanol by ultrasonication and then deposited onto the grid before drying them for observations.

### 2.8. Raman Spectroscopy

Raman analysis of GO, GO-AgNPs, not imprinted film (NIF@GO-AgNPs) and imprinted film (MIF@GO-AgNPs) before and after washing were performed by using a Senterra confocal Raman microscope (Bruker, Milan, Italy) with a laser excitation wavelength of 532 nm, 5 mW of nominal power, and a 100× objective. The spectra were recorded in the 70–4500 cm^−1^ range, with a resolution of 9 cm^−1^, an integration time of 3 s, and 6 co-additions.

### 2.9. Spectroscopic Ellipsometry (SE)

A α spectroscopic ellipsometer (J. A. Wollam, Lincoln, NE, USA.) with fixed angle geometry was used for measuring the thickness of the film. A Bruggeman effective medium approximation model with two components (void and Cauchy film) was used for fitting the experimental data. The refractive index parameters for the Cauchy film model were measured on reference samples made by the matrix and not containing neither the templating agent nor the Rh6G. Plots of Ψ and Δ as a function of the incident wavelength from 400 to 900 nm were simulated using the “CompleteEASE v. 4.2” program from Wollam. The results of the fits were evaluated on the basis of the mean squared error (MSE), which was maintained lower than 5.

### 2.10. Enhancement Efficiency and Molecular Selectivity of MI-G-SERS Platform

The evaluation of the Raman enhancement was obtained for all the not imprinted and molecularly imprinted films with and without the presence of the GO-Ag nanocomposite. Rh6G was used as a detection probe. 10 µL of a 1 × 10^−4^ M Rh6G ethanol solution was deposited onto the films and left to dry at 25 °C. A laser excitation wavelength of 785 nm, 1 mW of nominal power, and a 100× objective was used for Raman measurements. After each measurement, the samples were “recycled” by washing them with ultrasonication in ethanol to remove the residue of Rh6G. To investigate the molecular selectivity of the MI-G-SERS platforms towards structural analogues of the Rh6G, the Raman enhancement of the molecularly imprinted films containing the GO-Ag nanocomposite (MIF@GO-AgNPs) were analysed by depositing onto them same volumes of methylene blue (MB), Rhodamine B (RhB), and crystal violet (CV) having the same concentration of Rh6G.

### 2.11. Reproducibility of the MI-G-SERS Platform

The Raman measurement was performed by depositing 10 µL of a 1 × 10^−4^ M Rh6G solution onto the imprinted film containing the graphene oxide-AgNPs systems (MIF@GO-AgNPs). After each measurement, the films were washed with ethanol to remove the residues of Rh6G before being re-used. The molecularly imprinted film was washed by ultrasonication in ethanol and the complete removal of Rh6G traces was recorded by UV-Vis spectra. This process was repeated 10 times.

## 3. Results and Discussion

Despite the very promising results recently achieved in the field [28,30], the broad diffusion of GERS-active materials as smart platforms for the detection of specific analytes has been so far hampered by the low enhancement factor. This issue has raised the need to increase the sensitivity of these materials by moving from GERS to G-SERS based platforms. The latter, in fact, are capable of pairing the characteristic of graphene-related materials (embedding exfoliated graphene, graphene oxide, or reduced graphene oxide) with the sensitivity of SERS-based substrates (Ag, Au, or Cu nanoparticles). Moreover, if the material has to be used as a sensing platform, the selectivity and reusability also play important roles.

**Scheme 1:**
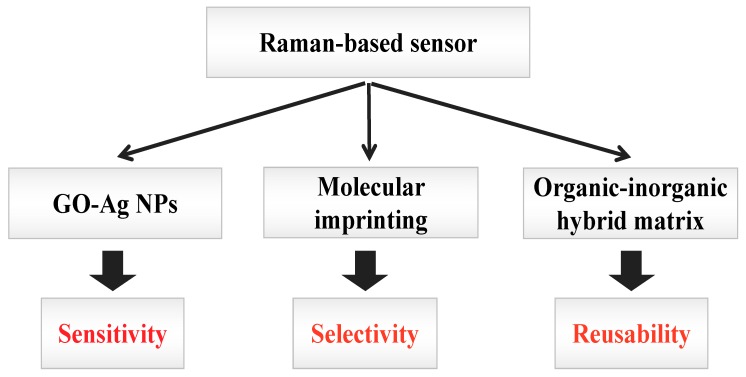
The designs of a Raman-based sensor that can combine sensitivity, selectivity, and reusability.

In this work, we have developed a Raman-based sensor with high sensitivity as well as molecular recognition capability and reusability. We have therefore designed the sensor by keeping in mind all these requirements that must be fulfilled (Scheme 1). The selectivity has been achieved by molecular imprinting and the sensitivity by combining GERS with SERS. The sensing platform has therefore been engineered by embedding a GO-Ag nanocomposite (GO-Ag NPs) into a molecularly imprinted organic-inorganic hybrid matrix.

GO-AgNPs has been prepared via a one-pot process by reducing AgNO_3_ in the presence of GO. The UV-Vis spectra show two bands at 240 and 402 nm assigned to the π-π* transition of GO and the plasmon of silver nanoparticles, respectively (Figure S1). The TEM images display spherical-like Ag NPs that are homogeneously loaded on the surface of the GO sheets with a dimension of around 8 nm (Figure S2).

The design of the molecularly imprinted hybrid platform has been obtained via a liquid phase approach with a careful selection of the precursors.

**Figure 1:**
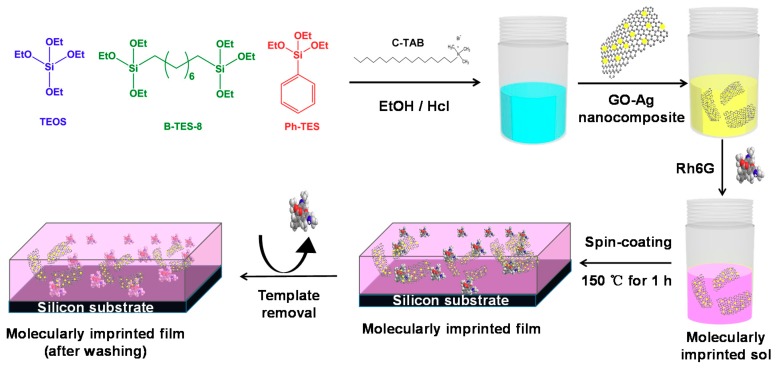
Schematic illustration of the preparation of a molecularly imprinted film containing graphene oxide Silver nanoparticles (MIF@GO-AgNPs). Tetraethylorthosilicate (TEOS), phenyltriethoxysilane (PhTES) and bis(triethoxysilyl)octane (B-TES-8) precursors build up the silica hybrid matrix embedding graphene oxide Silver nanoparticles (GO-AgNPs). Rhodamine 6G (Rh6G) acts as a molecular template; the surfactant cetyltrimethylammonium bromide (CTAB) provides the porosity by self-assembly. The film is deposited via spin-coating and removal of Rh6G and CTAB has been obtained by using ethanol to wash the samples.

The rationale of the synthesis is shown in Figure 1. We have produced molecularly imprinted films via a sol-gel process using Rh6G to imprint molecular cavities and CTAB, a surfactant molecule that can be easily removed, as a structure directing agent to create porosity in the matrix.

TEOS has been chosen to form the silica network, and B-TES-8 to increase the matrix flexibility and allow easy removal of the molecular template without losing any mechanical stability. Moreover, the phenyl groups in Ph-TES facilitate the homogeneous dispersion of GO-AgNPs into the matrix. After film deposition and drying, the template removal can be achieved by ultrasonication in ethanol, leaving free molecular cavities imprinted by Rh6G.

To understand the contributions that participate in the Raman enhancement, four types of hybrid films have been prepared: Not imprinted (NIF-H), molecularly imprinted (MIF-H), not imprinted with GO-AgNPs (NIF@GO-AgNPs), and molecularly imprinted with GO-AgNPs (MIF@GO-AgNPs).

**Figure 2:**
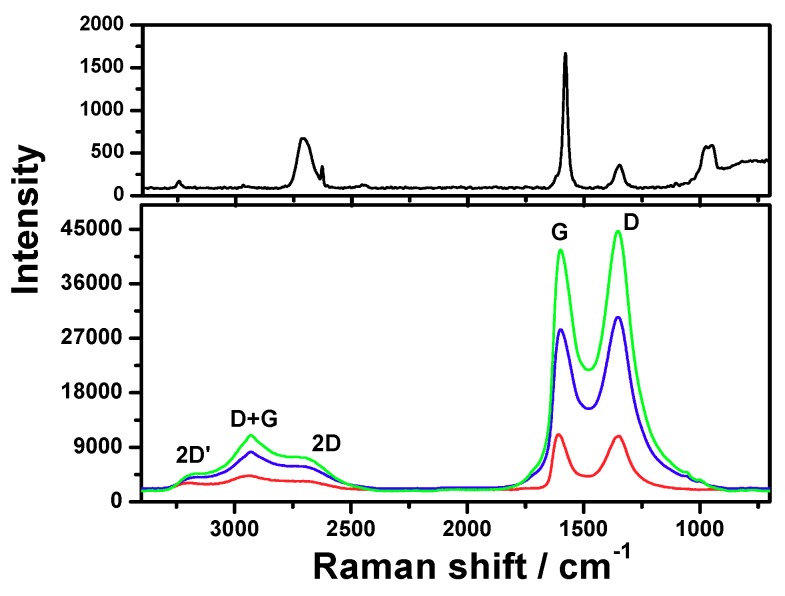
Raman spectra of graphite (top figure); GO (red curve), GO-AgNPs (green curve), and NIF@GO-AgNPs (blue curve) in the 3400–700 cm^−1^ range.

Figure 2 shows the Raman spectra of graphite, GO, GO-AgNPs, and not imprinted film embedding GO-AgNPs (NIF@GOAgNPs). The 2D, D + G, and 2D’ bands peaking at 2686, 2938, and 3205 cm^−1^, respectively, are clearly observed (Figure 2, bottom). Two intense bands at 1602 and 1350 cm^−1^, related to the breathing mode of k-point phonons of A_1g_ symmetry (G band) and attributed to the E_2g_ phonon of sp^2^ carbon atom (D band), are also detected [32]. Compared to graphite, the GO shows a higher I_D_/I_G_ ratio, suggesting the successful formation of GO sheets from graphite [33]. The I_D_/I_G_ ratio is generally correlated to the number of defects and disorder on the GO surface [34,35]. The I_D_/I_G_ ratios of GO, GO-AgNPs, and NIF@GO-AgNPs (Figure 2) are 0.96, 1.07, and 1.12, respectively. The I_D_/I_G_ ratio of GO-AgNPs is higher than in the GO spectra, indicating a larger number of defects and disorder within the material. This suggests that AgNPs are a source of defect formation on the GO surface. After embedding GO-AgNPs into the hybrid matrix, the I_D_/I_G_ decreases from 1.12 to 1.07; the small decrease shows that the embedding process does not affect the surface properties of GO. In addition, the Raman intensity of GO-AgNPs and NIF@GO-AgNPs Raman spectra is much higher compared to the bare GO Raman spectra, indicating that the AgNPs increase the GO Raman signal due to the electromagnetic enhancement of AgNPs [26].

### 3.1. Molecular Imprinting and Template Removal

**Figure 3:**
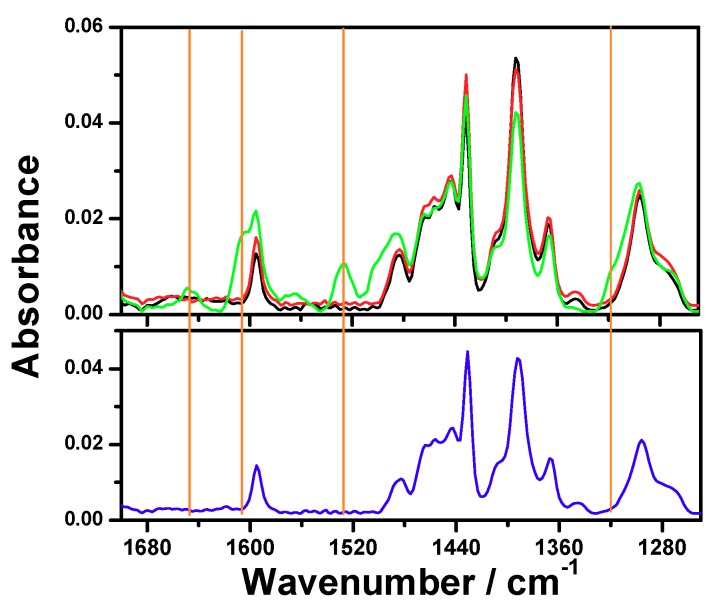
FTIR spectra of NIF-H (black curve), NIF@GO-AgNPs (red curve), and MIF@GO-AgNPs samples before (green curve) and after washing (blue curve, bottom figure).

Figure 3 shows the NIF-H, NIF@GO-AgNPs, and MIF@GO-AgNPs FTIR spectra in the 1700–1250 cm^−1^ range (the same spectra in the 4000–400 cm^−1^ range are shown in Figure S3). The main bands of Rh6G at 1319, 1525 and 1605 cm^−1^, corresponding to the aromatic C-C stretching vibrational modes, can be clearly observed in the imprinted film before washing (green curve). This confirms the successful imprinting of the hybrid film.

On the contrary, after washing the films with ethanol, the Rh6G bands disappear (blue curve), indicating that the template can be completely and easily removed after imprinting.

**Figure 4:**
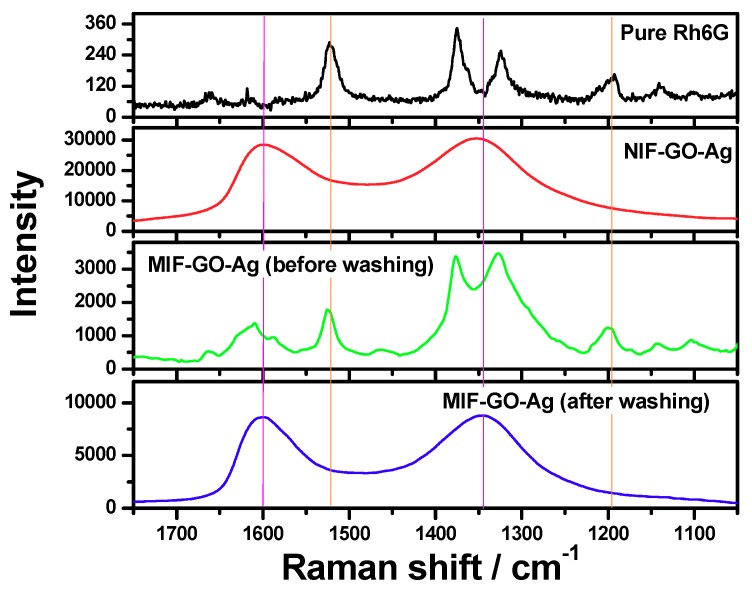
Raman spectra of pure Rh6G (20 µL, 10^−3^ M) deposited on a silicon substrate (black curve), NIF@GO-AgNPs (red curve), and MIF@GO-AgNPs before (green curve) and after (blue curve) washing.

These results have been also confirmed by Raman spectroscopy (Figure 4) using the Rh6G signal as a reference (top Figure 4). MIF@GO-AgNPs before washing (green curve) shows the characteristic D band and G bands of GO (1602 and 1350 cm^−1^) and the typical Rh6G bands (1525 and 1196 cm^−1^), indicating that the imprinted film contains both the GO-AgNPs and the molecular template. The G band in the NIF@GO-AgNPs peaks at 1602 cm^−1^, while in MIF@GO-AgNPs it shifts to 1610 cm^−1^ due to the strong interaction between the molecularly imprinted sites and GO-AgNPs [36]. After ultrasonicating the NIF@GO-AgNPs sample in ethanol (blue curve), the D and G bands are still visible while the Rh6G bands disappear. This means that the template molecule can be completely removed from the imprinted film without affecting the GO-AgNPs in the matrix. Additionally, the same I_D_/I_G_ ratios of MIF@GO-Ag (after washing) and NIF@GO-AgNPs indicate that the imprinted sites do not affect the GO surface. It is important to stress that Figure 4 only highlights the difference in the Raman spectra of the MIF@GO-AgNPs film before and after Rh6G is removed from the matrix. The spectra, in fact, have been collected by using a Raman microscope focused onto a graphene flake embedded into the hybrid matrix. However, while optimizing the measure of the Rh6G deposited onto the hybrid surface, the signal coming from the GO flake, which is embedded deeper into the matrix, is far from being optimized and thus shows a lower intensity that can be easily covered by both the Rh6G and matrix. Under these conditions, the peak intensity of the GO cannot be quantitatively compared between the spectra (green curve and blue curve) because, as explained above, the difference in focus depth results in different intensities.

Surface morphology, area, and thickness can strongly affect the Raman enhancement performance when used as a sensing platform [13,14]. The thicknesses of NIF-H, MIF-H, NIF@GO-AgNPs, and MIF@GO-AgNPs samples have been estimated as 1140, 1055, 1089, and 963 nm, respectively (See Table S1 in Supplementary Materials). After washing, the thickness decreases by 8% and the porosity of both the imprinted and not imprinted films increased by 3.4%.

**Figure 5:**
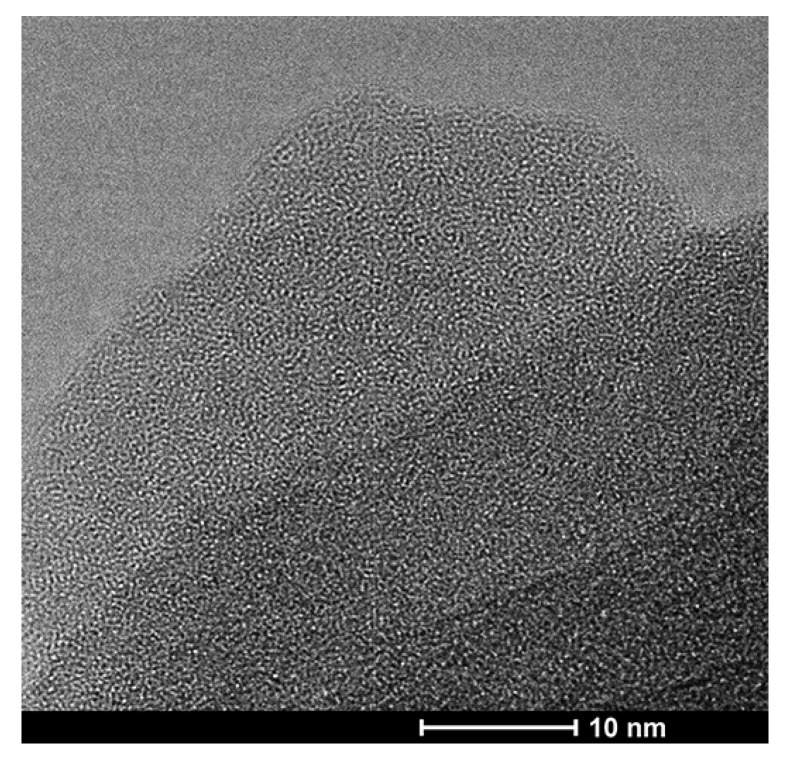
TEM image of the hybrid matrices (sample not imprinted and without GO/Ag NPs).

The TEM characterization of the hybrid matrices (samples not imprinted and without GO/Ag NPs) shows the formation of a microporous structure with a poor degree of organization (wormlike) and an average pore size smaller than 1 nm, which is in agreement with our previous findings (Figure 5) [30].

The free cavities in the molecularly imprinted film provide the recognition property; a complete removal of the template molecule is, however, a very critical stage. The imprinted material should be flexible enough to allow easy removal while preserving the structural integrity of the imprinted cavities. The conventional removal method for non-covalent imprinting is to disrupt the week electrostatic forces holding the template inside the cavities by using some organic reagents, buffer solutions, or even acidic treatments [26,27]. A full removal of the template maximizes the number of imprinted cavities that are available for the molecular recognition. Moreover, the quantification of the imprinted cavities is also very important to evaluate the efficiency of the signal and to understand the interplay between the molecular recognition and the performance of the Raman enhancement.

**Figure 6:**
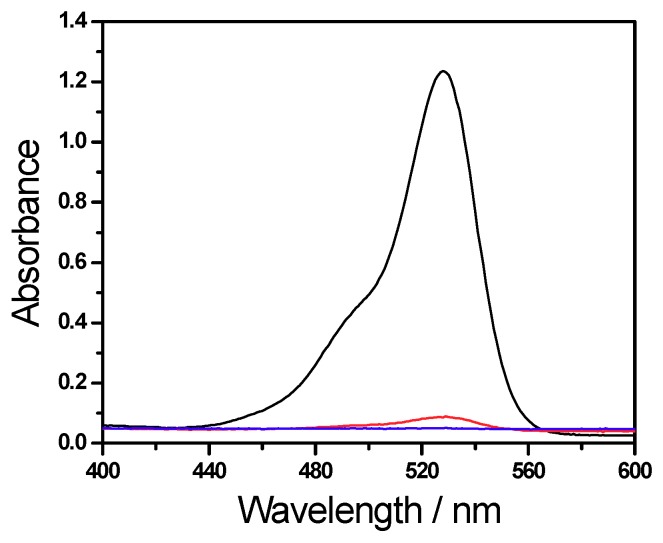
UV-Vis spectra of the ethanol solutions used for washing the MIF@GO-AgNPs sample (black curve for first washing, red curve for the second washing, and blue curve for the third washing).

UV-Vis spectroscopy has been used to assess the number of molecular cavities in each imprinted film as previously described [28,30]. The ethanol solution after the first washing cycle shows a strong absorption band peaking at 528 nm (Figure 6), which suggests that most of the template molecules (90%) have been removed. The third washing solution does not show any absorption band that could be correlated with the residual presence of Rh6G, confirming that the template molecule has been entirely removed from the MIF@GO-AgNPs matrix. A similar procedure has also been used to empty the molecular cavities in NIF-H and MIF-H, obtaining very similar results (Figure S4).

The molar extinction coefficient at 528 nm has been calculated by the Lambert-Beer law to be 118,900 L·mol^−1^·cm^−1^. The total concentration of Rh6G washed out from the imprinted film has been estimated in 2.182 × 10^−4^ mmol. This value, divided by the washing volume (9 mL) of the imprinted film, allows the density of molecular cavities present into a MIF@GO-AgNPs sample to be evaluated as 3.77 × 10^−10^ µmol·µm^−3^. This estimate is the maximum number of imprinted cavities per film volume, since not all the imprinted cavities can be considered as fully active. Therefore, the real number of imprinted active cavities should be lower than the calculated value. The number of molecular cavities can be also estimated by considering the Rh6G concentration in the gel and supposing that Rh6G is completely removed from the MIF-H sample. Following these assumptions, the density of imprinted cavities can be calculated as 4.75 × 10^−10^ µmol·µm^−3^.

### 3.2. Raman Enhancement Performance of MI-G-SERS Sensing Platform

**Figure 7:**
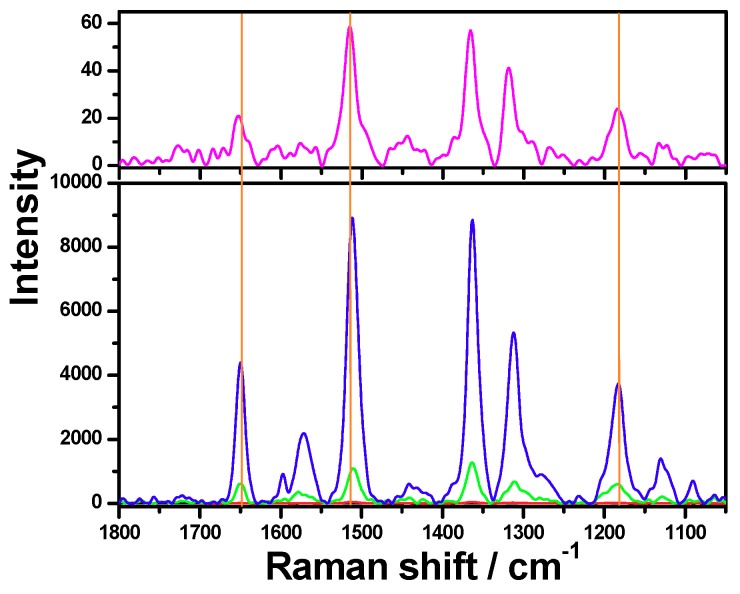
Raman spectra of Rh6G (1 × 10^−4^ M) deposited onto a silicon substrate (top magenta curve), NIF-H (black curve), MIF-H (red curve), NIF@GO-AgNPs (green curve), and MIF@GO-AgNPs sample (blue curve).

To investigate the Raman enhancement, we have deposited the same volume of Rh6G solution (10 µL, 1 × 10^−4^ M) onto NIF-H, MIF-H, NIF@GO-AgNPs, and MIF@GO-Ag samples. Figure 7 shows the Raman spectra obtained from each film; the pure Rh6G solution deposited on a silicon substrate is used as a reference (top). The Raman bands of Rh6G detected at 1650, 1510, 1363, and 1312 cm^−1^ have been assigned to the aromatic C-C stretching, while the band at 1182 cm^−1^ has been attributed to C-O-C. The MIF-H and NIF@GO-AgNPs samples display a higher enhancement effect with respect to the NIF-H sample. Moreover, the MIF@GO-AgNPs sample) shows a much higher Raman intensity of Rh6G in comparison with the not imprinted (NIF-H and NIF@GO-AgNPs) and the imprinted film without GO-AgNPs (MIF-H).

**Figure 8:**
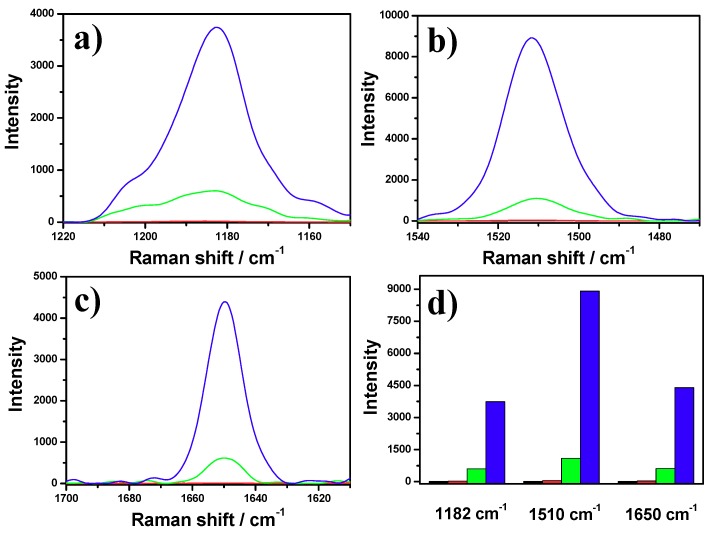
Raman spectra of Rh6G (1 × 10^−4^ M) deposited onto NIF-H (black curve), MIF-H (red curve), NIF@GO-AgNPs (green curve), and MIF@GO-AgNPs sample (blue curve) related to three Raman modes centered at 1182 cm^−1^ (**a**), 1510 cm^−1^ (**b**), and 1650 cm^−1^ (**c**). (**d**) Bar plot comparing the signal intensities obtained from NIF-H (black bar), MIF-H (red bar), NIF@GO-AgNPs (green bar), and MIF@GO-AgNPs (blue bar) taken for the different Raman modes.

The Rh6G Raman modes at 1650, 1510, and 1182 cm^−1^ have been selected to analyze the enhancement performance. Different Raman modes exhibit a different response in terms of relative intensities due to the “chemical enhancement” mechanism due to a charge transfer between the analyte and the graphene substrate [5,37]. MIF@GO-AgNPs provides the highest intensities of Rh6G (Figure 8). The Raman intensities follow this trend: MIF@GOAgNPs > NIF@GOAgNPs > MIF-H > NIF-H.

To better visualize the enhancement provided by these sensing platforms, we have compared the intensities of Rh6G bands at 1650, 1510, and 1182 cm^−1^. All the reported intensities of Rh6G in the bar plot of Figure 8d are the average of seven measurements taken at different points of each film. The NIF@GO-AgNPs sample shows a 68.5-fold increase of the Raman intensity with respect to the NIF-H substrate. The Raman intensity enhancement provided by the MIF@GO-AgNPs sample is 27.4 times higher than that one measured in a molecularly imprinted film embedding only exfoliated graphene (MI-GERS) (2.5–fold). Compared to the NIF-H sample, the intensity obtained from the MIF-H sample displays a 3.4-fold enhancement, which is due to the imprinted cavities. Moreover, the MIF@GO-AgNPs substrate exhibits an 8.2–fold enhancement in comparison to NIF@GO-AgNPs. The Rh6G Raman band at 1510 cm^−1^ of MIF@GO-AgNPs shows, on average, a signal that is 557 times more intense than that measured on an NIF-H substrate. This remarkable Raman enhancement observed for MIF@GO-AgNPs can be attributed to a cooperative effect between the molecularly imprinted cavities and the GO-AgNPs.

The analytical enhancement factor (AEF) has been calculated using the following formula [38]:

(1) $$AEF=\frac{I_{ERS}}{I_{NIF-H}}\times \frac{C_{NIF-H}}{C_{ERS}}\;$$

*I_ERS_* are the Raman intensities due to the enhanced Raman scattering of ERS-active substrates measured at 1650, 1510, and 1182 cm^−1^ (Rh6G modes). The film without GO-AgNPs and imprinted cavities (*I_NIF–H_*) has been chosen as the reference substrate since it does not show any Raman enhancement activity.

*C_ERS_* and *C_NIF-H_* are the corresponding concentrations of Rh6G deposited on the films. The AEF depends on all the adsorbed molecules on the films under the laser spot area. The concentration of Rh6G molecules adsorbed onto the film can therefore be estimated as Equation (2):

(2) $$C=\frac{n}{s\cdot h\cdot p_{}}=\frac{10^{-4}\cdot V_{Rh6G}}{s\cdot h\cdot p}\;$$

The *V_Rh6G_* is the constant volume (10 µL) of the Rh6G solution (*C* = 10^−4^ M) deposited on the films for Raman measurements; *s* is the spot size of the Raman laser, which is equal to 0.9642 µm^2^; *h* is the thickness of each film; and *p* is the porosity of the films. The porosities of each film, obtained by spectroscopic ellipsometry, are almost the same and are around 3.4%. Therefore, assuming that the porosity of all the samples is comparable, the increase of surface area provided by the imprinted cavities is negligible. Since the *V_Rh6G_* and laser spot size (*s*) are constant, Equation (3) can be simplified as:

(3) $$AEF=\frac{I_{ERS}}{I_{NIF-H}}\times \frac{h_{NIF-H}}{h_{ERS}}\;$$

**Figure 9:**
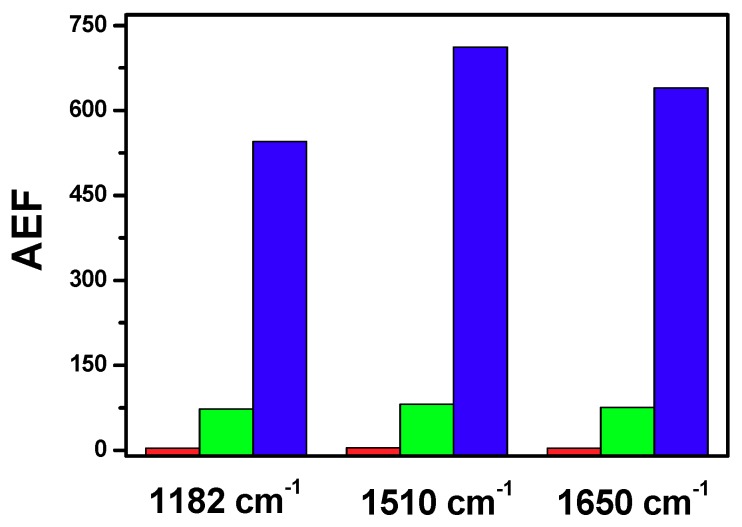
Bar plot of analytical enhancement factor (AEF) of MIF-H (red bar), NIF@GO-AgNPs (green bar), and MIF@GO-AgNPs (blue bar) at different Raman modes, 1182, 1510, and 1650 cm^−1^.

The AEF results with different Raman modes are reported in Figure 9. By comparing the AEF of NIF@GO-AgNPs, MIF-H, and MIF@GO-AgNPs samples, three different enhanced Raman scattering contributions have been investigated: The GO-AgNPs enhanced Raman scattering activity (G-SERS), the molecularly imprinted activity (MI), and the cooperate efficiency of the GO-Ag nanocomposite and imprinted cavities.

The activity of the imprinted cavities, in fact, gives a molecular recognition capability, which leads to the AEFs of MIF-H having a 3.5-fold increase at 1182 cm^−1^, a 4-fold increase at 1510 cm^−1^, and a 3.6-fold increase at 1650 cm^−1^. The embedding of GO-AgNPs allows a 73-fold increase of the signal at 1182 cm^−1^, 81-fold increase at 1510 cm^−1^, and a 76-fold increase at 1650 cm^−1^. This enhancement should be mainly attributed to the combination of a strong EM enhancement from Ag particles and a CM enhancement due to the interaction between the GO surface and Rh6G. In the case of MIF@GO-AgNPs, the AEFs show a 545-fold enhancement at 1182 cm^−1^, a 712-fold enhancement at 1510 cm^−1^, and a 640-fold enhancement at 1650 cm^−1^. These enhancements can be explained by considering the interplay between molecularly imprinted cavities and the embedded GO-AgNPs. In summary, the MIF@GO-AgNPS sample allows a 712-fold AEF to be obtained, which is much higher than in previous reports. Moreover, this is, to the best of our knowledge, the highest AEF for Rh6G related to G-SERS-active platforms [28].

To evaluate the recognition effect provided only by the molecular imprinting approach [28,30], we have estimated an effect related to the imprinted cavities by using the efficiency of the imprinted film (MIF) with respect to the not imprinted film (NIF). We have assumed that all of the imprinted cavities in the films could contribute to the enhancement effect calculated by the following Equation (4):

*I_cavity_* = *I_MIF_* − *I_NIF_* (4)

Then *I_cavity_* has been divided by *N_cavity_* (number of cavities) to obtain the enhancement per single cavity (*EF_cavity_*) illuminated by the laser focus spot. The laser spot area is 0.9642 µm^2^ and the film thickness is around 1 µm; therefore, the laser spot volume (LSV) is 0.9642 µm^3^. Thus, we can estimate the number of molecules illuminated by the laser as 3.64 × 10^−10^ (MIF@GO-AgNPs) and 4.588 × 10^−10^ (MIF-H) µmol, respectively. Now, we can calculate the *EF_cavity_* using the Equation (5) as follows:

(5) $$EF_{cavity}=\frac{I_{cavity}}{N_{cavity}}\;$$

By considering the intensity of Rh6G at 1510 cm^−1^ and the number of cavities within the laser spot volume, the *EF_cavity_* value is respectively equal to 2.15 × 10^13^ (MIF@GO-AgNPs) and 8.28 × 10^10^ count/µmol (MIF-H). It is important to highlight that this value depends upon the Raman mode and, as shown in Figure S5, the Raman mode at 1510 cm^−1^ gives the higher response. It should also be considered that the *EF_cavity_* value is an overestimate since we have assumed that all the cavities from the MIF sample illuminated by the Raman laser have been efficiently imprinted.

The results indicate that a limited number of molecular cavities could provide a quite remarkable amplification of Raman enhancement, which is further increased by the presence of GO-AgNPs. This increase can be attributed to the synergistic effect of G-SERS and molecular imprinting, which, at least to some extent, can amplify the detection of the molecules trapped into the molecular cavities. In addition, the *EF_cavity_* value of our present platform increases by 7.5 times in comparison to the MI-GERS platform.

### 3.3. Reproducibility and Selectivity

**Figure 10:**
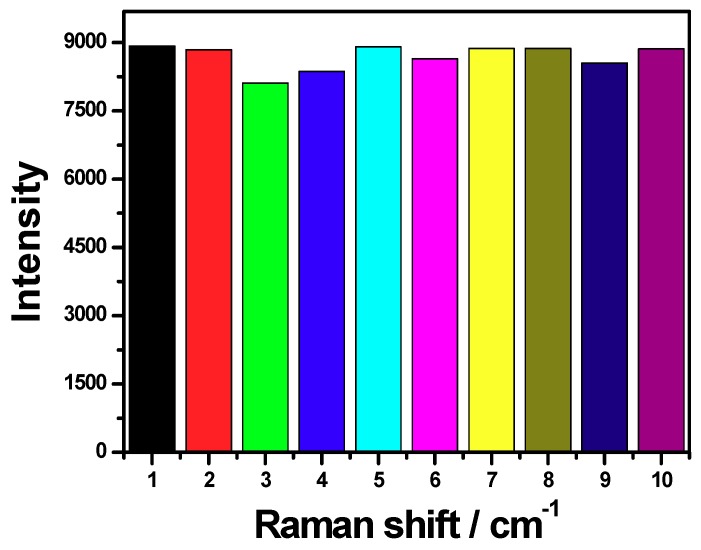
Reusability of MIF@GO-AgNPs substrates for Rh6G detection, measured by the changes in the intensity of the 1510 cm^−1^ Raman band.

Reusability is an important property of any sensor as it allows testing the reproducibility of the detection. In general, a GO-AgNPs material deposited onto a substrate cannot be reused for a second detection. In the case of our MI-G-SERS platform, after each measurement, the target molecule can be easily removed from the film using ethanol. We have performed a cycle-dependent Raman enhancement measurement for investigating the reproducibility of the MI-G-SERS platform. Figure 10 shows the intensities of Rh6G obtained after each washing cycle of the MIF@GO-AgNPs substrate. The results show excellent reproducibility, with an average standard deviation of the intensities smaller than 6%. After 10 times recycling, the MIF@GO-AgNPs systems do not lose efficiency, indicating that the washing process efficiently removes all Rh6G without degrading the platform.

**Figure 11:**
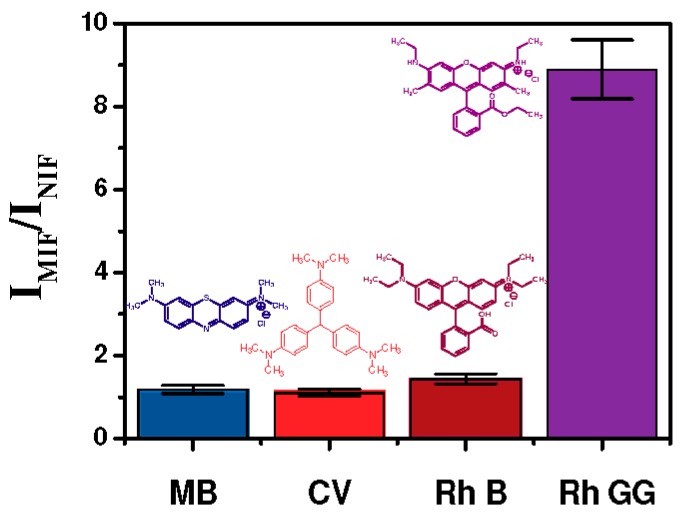
Selectivity of MIF@GO-AgNPs substrate towards solutions of methylene blue (MB, 1393 cm^−1^ Raman mode), crystal violet (CV, 1375 cm^−1^ Raman mode), rhodamine B and 6G (RhB and Rh6G, 1510 cm^−1^ Raman mode) with respect to the NIF@GO-AgNPs substrate.

The selectivity of the sensor is also a pivotal feature that should be considered. This property has been tested using other organic dyes: Rhodamine B (RhB), methylene blue (MB), and crystal violet (CV). Figure 11 shows a bar plot with the Raman band intensities of the different dyes measured on the MIF@GO-AgNPs substrates divided by their corresponding intensities on the NIF@GO-AgNPs substrates (*I*_MIF@GO-AgNPs_/*I*_NIF@GO-AgNPs_). It is observed that the Raman enhancement for Rh6G from the MIF@GO-AgNPs substrate is much higher than that of the other three dyes (RhB, CV, and MB), suggesting that the MIF@GO-AgNPs substrate has a higher affinity for the template molecule (Rh6G). For Rh6G deposited onto the MIF@GO-AgNPs substrate, the obtained *I*_MIF@GO-AgNPs_/*I*_NIF@GO-AgNPs_ value is as high as 8.2, while for MB, CV, and RhB it is 1.47, 1.08, and 1.22, respectively. The *I*_MIF-GO-Ag_/*I*_NIF-GO-Ag_ value of Rh6G is significantly higher than those obtained from not imprinted molecules. The imprinted free cavities in the MIF@GO-AgNPs are very efficient to give a selective recognition of Rh6G molecules. In addition, the *I*_MIF@GO-AgNPs_/*I*_NIF@GO-AgNPs_ values of MB, CV, and RhB are close to 1, which means that there is almost no difference between the imprinted and not imprinted films. The weak enhancement is only due to physical adsorption onto the graphene oxide surface and not to molecular recognition. The highly specific recognition property is due to the imprinted cavities, which matched the size, shape, and functional group of Rh6G. Although RhB has a chemical structure similar to Rh6G and MB and CV bear similar functional groups (amino groups), the cavities cannot match them as tightly as Rh6G. The higher *I*_MIF@GO-AgNPs_/*I*_NIF@GO-AgNPs_ ratio observed in MB with respect to CV and RhB is due to the smaller steric hindrance, which allows the dye to randomly enter the molecular cavities.

## 4. Conclusions

Molecularly imprinted organic-inorganic hybrid films embedding graphene oxide sheets decorated with silver nanoparticles are highly selective and sensitive platforms for Raman sensing. The material has shown an excellent Raman enhancement due to the combined effect of graphene oxide, silver nanoparticles, and molecular cavities. A 712-fold analytical enhancement factor has been achieved corresponding to a Raman enhancement per single molecular cavity equal to 2.15 × 10^13^. The sensing platforms are reusable as they can withstand several detection cycles without losing efficiency. Finally, the films have shown a highly selective enhancement, being able to discriminate Rh6G, used as a probe molecule, with respect to other dyes with a similar chemical structure.

## Supplementary Materials

The following are available online at http://www.mdpi.com/1996-1944/11/9/1674/s1, Figure S1: UV-vis spectra of GO and GO-Ag nanocomposite, Figure S2: TEM images of GO-Ag nanocomposite and Ag particle size distribution of the GO-Ag, Figure S3: FTIR spectra of NIF-H, NIF@GO-AgNPs and MIF@GO-AgNPs before and after washing samples in the 400–4000 cm^−1^ range, Figure S4: UV-Vis spectra of the ethanol solutions used for washing the MIF-H sample, Figure S5: The effect factor of Rh6G obtained from MIF-H and MIF@GO-AgNPs calculated at different Raman modes, Table S1: Film thickness of NIF-H, MIF-H, NIF@GO-AgNPs and MIF@GO-AgNPs samples before and after CTAB removal.

## Appendix A

### Preparation of Graphene Oxide (GO)

1 g of graphite powder, 2.5 mL of H_3_PO_4_, and 23 mL of H_2_SO_4_ were mixed into a three-necked round-bottom flask. After vigorous stirring for 30 min, 12 g of KMnO_4_ was slowly added into the reaction container and then the mixture reacted at 50 °C for 6 h. 100 mL of water was then added drop-wise into the flask after waiting for 30 min, and the mixture was then poured into 500 mL of milli-Q water. 2 mL of H_2_O_2_ (27%) was then added drop-wise followed by vigorous stirring, and, finally, the mixture solution became gold yellow. Afterwards, the mixture was washed by using 5% HCl water solution and water for several times (each washing cycle, the supernatant was centrifuged at 10,000 rpm for 5 min) until the neutral pH of the solution was checked to ensure a complete removal of residual metal ions. Then, it was moved to 60 °C for 24 h to evaporate the water. At last, the GO powder was sonicated for 2 min and re-dispersed in ethanol.
