# Supplementary material for: Graphene Oxide-Silver Nanoparticles in Molecularly-Imprinted Hybrid Films Enabling SERS Selective Sensing
Source: Materials (Basel). 2018 Sep 10;11(9):1674. doi: 10.3390/ma11091674 (PMC6163847; doi:10.3390/ma11091674)
Supplement: Supplementary file 1 [file materials-11-01674-s001.pdf]

Supplementary Materials

Graphene oxide-silver nanoparticles in molecularly-imprinted hybrid films enabling SERS selective sensing

Y. Jiang[1], D. Carboni[1], L. Malfatti[1*] and P. Innocenzi[1*]

[1] Laboratorio di Scienza dei Materiali e Nanotecnologie, Dipartimento di Chimica e Farmacia, Università di Sassari, CR-INSTM, Via Vienna 2, Sassari 07041. Italy; jyu@uniss.it; dcarboni@uniss.it; luca.malfatti@uniss.it; plinio@uniss.it

* Correspondence: luca.malfatti@uniss.it; plinio@uniss.it; Tel.: +39-079-998630

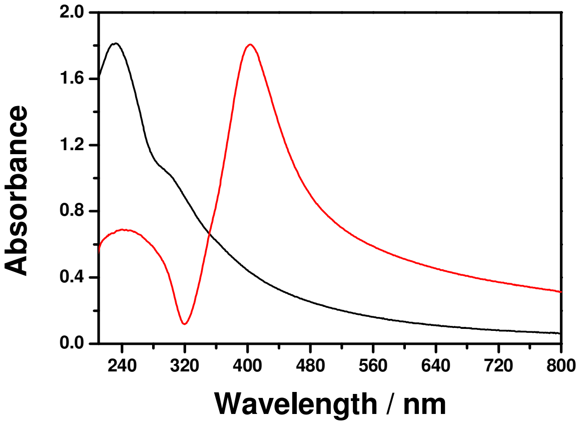

Figure. S1. UV-vis spectra of GO (black curve) and GO-Ag nanocomposite (red curve).

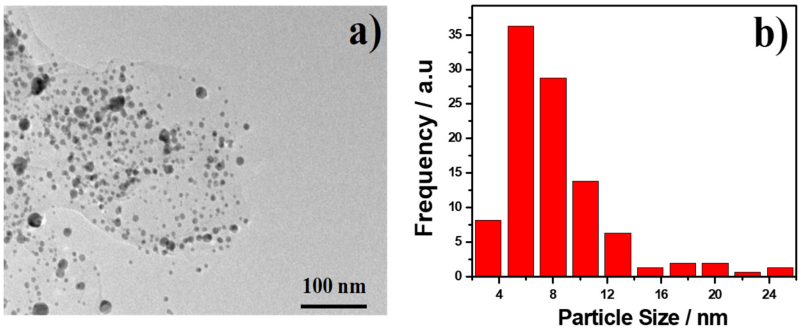

Figure. S2 (a)TEM images of GO-Ag nanocomposite. **(b)** Ag particle size distribution of the GO-Ag nanocomposite.

Materials 2018, *11*, 1674; doi:10.3390/ma11091674

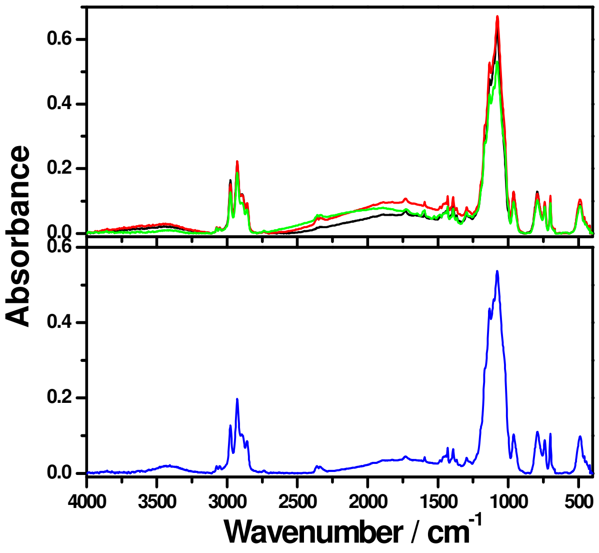

Figure. S3. FTIR spectra of NIF-H (black curve), NIF@GO-AgNPs (red curve) and MIF@GO-AgNPs before (green curve)and after washing samples in the 4000-400 cm^{-1} range (bottom figure blue curve, bottom figure).

The FTIR spectra of not imprinted hybrid film (NIF-H), not-imprinted hybrid film (NIF@GO-AgNPs) and imprinted hybrid films (MIF@GO-AgNPs) with GO-Ag nanocomposite, in the 4000-400 cm^{-1} range, are shown in **Fig. S3** of the supporting information. Two characteristic peaks at 964 and 3410 cm^{-1} are attributed to the Si-OH stretching and -OH stretching. One intensive signal peaked at 1080 cm^{-1} is assigned to the anti-symmetric stretching of the Si-O-Si silica band, providing the evidence of silicate network in the hybrid film. Furthermore, another band at 489 cm^{-1}, which can be ascribed to the Si-O-Si bending vibration. It is also noted that two overlapped bands at 1166 and 1134 cm^{-1} can be observed, which refer to the signature of unreacted or uncondensed silica, can be observed.

Materials 2018, 11, 1674; doi:10.3390/ma11091674

Samples	Before template removal / (nm)	After template removal / (nm)
NIF-H	1140 ± 3	1056 ± 5
MIF-H	1055 ± 4	965 ± 6
NIF-GO-Ag	1089 ± 10	1007 ± 11
MIF-GO-Ag	963 ± 13	892 ± 15

Table. S1. Film thickness of NIF-H, MIF-H, NIF@GO-AgNPs and MIF@GO-AgNPs samples before and after CTAB removal.

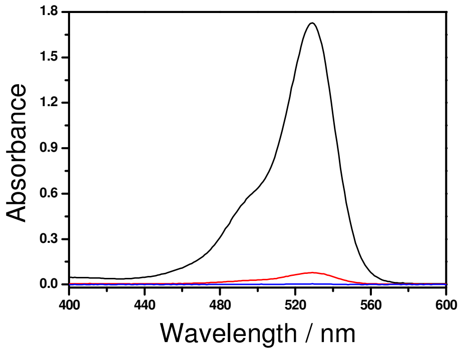

Figure. S4. UV-Vis spectra of the ethanol solutions used for washing the MIF-H sample (black curve for first washing, red curve for the second washing and blue curve for the third washing).

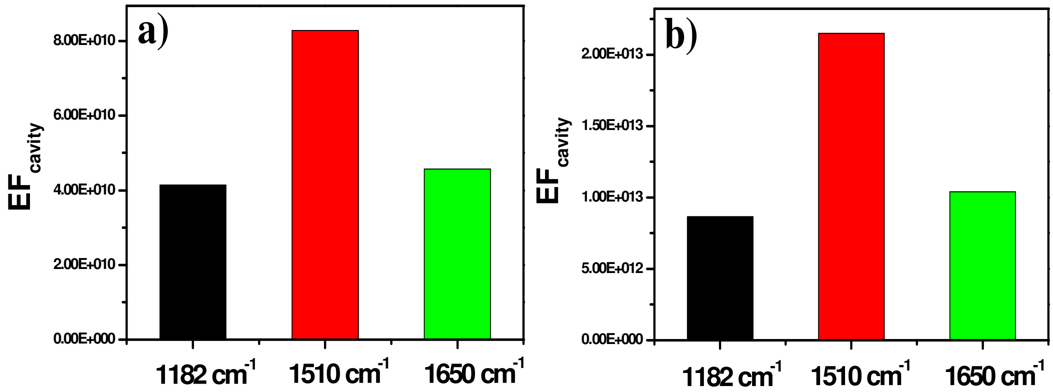

Figure. S5. The effect factor (EF) defines the signal enhancement per single cavity of Rh6G obtained from MIF-H (**a**) and MIF@GO-AgNPs (**b**) taken for the different Raman modes.

Materials 2018, 11, 1674; doi:10.3390/ma11091674
